# The success factors of scaling-up Estonian sexual and reproductive health youth clinic network - from a grassroots initiative to a national programme 1991–2013

**DOI:** 10.1186/1742-4755-12-2

**Published:** 2015-01-08

**Authors:** Jari Kempers, Evert Ketting, Venkatraman Chandra-Mouli, Triin Raudsepp

**Affiliations:** Qalys Health Economics, Amsterdam, the Netherlands; Radboud University Nijmegen Medical Centre, Nijmegen, the Netherlands; Department of Reproductive Health and Research, World Health Organization, Geneva, Switzerland; Estonian Sexual Health Association, Tallinn, Estonia

**Keywords:** Adolescents, Youth-friendly, Youth clinic, Sexual and reproductive health, Scale-up, Framework

## Abstract

**Background:**

A growing number of middle-income countries are scaling up youth-friendly sexual and reproductive health pilot projects to national level programmes. Yet, there are few case studies on successful national level scale-up of such programmes. Estonia is an excellent example of scale-up of a small grassroots adolescent sexual and reproductive health initiative to a national programme, which most likely contributed to improved adolescent sexual and reproductive health outcomes. This study; 1) documents the scale-up process of the Estonian youth clinic network 1991–2013, and 2) analyses factors that contributed to the successful scale-up. This research provides policy makers and programme managers with new insights to success factors of the scale-up, that can be used to support planning, implementation and scale-up of adolescent sexual and reproductive health programmes in other countries.

**Methods:**

Information on the scale-up process and success factors were collected by conducting a literature review and interviewing key stakeholders. The findings were analysed using the WHO-ExpandNet framework, which provides a step-by-step process approach for design, implementation and assessment of the results of scaling-up health innovations.

**Results:**

The scale-up was divided into two main phases: 1) planning the scale-up strategy 1991–1995 and 2) managing the scaling-up 1996–2013. The planning phase analysed innovation, user organizations (youth clinics), environment and resource team (a national NGO and international assistance). The managing phase examines strategic choices, advocacy, organization, resource mobilization, monitoring and evaluation, strategic planning and management of the scale-up.

**Conclusions:**

The main factors that contributed to the successful scale-up in Estonia were: 1) favourable social and political climate, 2) clear demonstrated need for the adolescent services, 3) a national professional organization that advocated, coordinated and represented the youth clinics, 4) enthusiasm and dedication of personnel, 5) acceptance by user organizations and 6) sustainable funding through the national health insurance system. Finally, the measurement and recognition of the remarkable improvement of adolescent SRH outcomes in Estonia would not have been possible without development of good reporting and monitoring systems, and many studies and international publications.

## Background

A small but growing number of middle-income countries are scaling up pilot projects on youth-friendly sexual and reproductive health (SRH) services, to district, province and national level programmes. Yet, there are few case studies or publications on successful national level scale-up of such programmes. This study: 1) documents the scale-up process of the Estonian Youth Clinic Network (YCN) during the period 1991–2013, and 2) analyses factors that contributed to the successful scale-up of the YCN, by using the WHO-ExpandNet framework [1–3]. This research provides policy makers and programme manager with new information of the factors that contributed to the successful national level scale-up of YCN in Estonia. The information can be used to support planning, implementation and scale-up of adolescent SRH programmes in other countries. This report builds on the World Health Organizations’ (WHO) case study of the Estonian YCN [4].

Estonia is the northernmost of the three Baltic States. The population is 1.3 million, of which 12% (155,000 persons) are 15–24 years [5]. 70% is Estonian speaking and one quarter Russian speaking. In 1991 Estonia regained its independence from the Soviet Union. In 2004 the country became a member of the European Union and adopted the Euro currency in 2011.

Estonia is an excellent example of scaling up a small grassroots SRH initiative to a national YCN, which most likely contributed to improved adolescent SRH outcomes. The first youth clinic (YC) was started in 1991. During the following decade the YCN was successfully scaled up to a national network of 18 YCs, which is coordinated and represented by the Estonian Sexual Health Association (ESHA). SRH outcomes of Estonian youth improved remarkably during the period 2001–2009. Annual rates of abortions, STIs and diagnosed HIV infections among 15–24 year olds were reduced by 37%, 55% and 89%, respectively [6, 7]. The YCN was implemented simultaneously with the Estonian school-based sexuality education (SBSE) programme [8]. Together the two interventions have most likely contributed to the improvement of SRH outcomes [7].

## Methods

A systematic search was conducted to identify relevant literature on the Estonian YCs. A snowball sampling technique, i.e. by reviewing references and other publications of selected authors was used to identify more publications, including grey literature. The initial literature review showed that most publications were written from the perspective of the YCN. Therefore, additional information was collected from two complementary perspectives by interviewing: 1) Dr Tiiu Aro, a former Minister of Social Affairs of Estonia (MSA) 1996–1999, who provided a policy makers point of view, and 2) Dr Sirje Vaask who gave insights in the development of the Estonian Health Insurance Fund (EHIF) and its relationship with the YCs. Moreover, the authors were already familiar with the YCN through their previous work.

The scale-up process and success factors of the Estonian YCN were analysed with the WHO-ExpandNet framework, which provides a step-by-step process approach for design, implementation and assessment of the results of scaling-up health innovations [1]. It contains two complementary sections. First, planning the scale-up strategy, which covers four key elements: innovation to be scaled up, user organizations, environment and resource organization. Second, managing the scaling-up, which has two subsections: 1) organization and management, which includes: strategic choices for scaling up, advocacy, organizing the scaling-up process, mobilization of resources, and monitoring and evaluation, and 2) strategic planning and management of scale-up. This study is a retrospective assessment of the YCN scale-up process applying the WHO-ExpandNet framework. The framework was not used in planning the YCN scale-up in Estonia.

## Results

**Figure 1:**
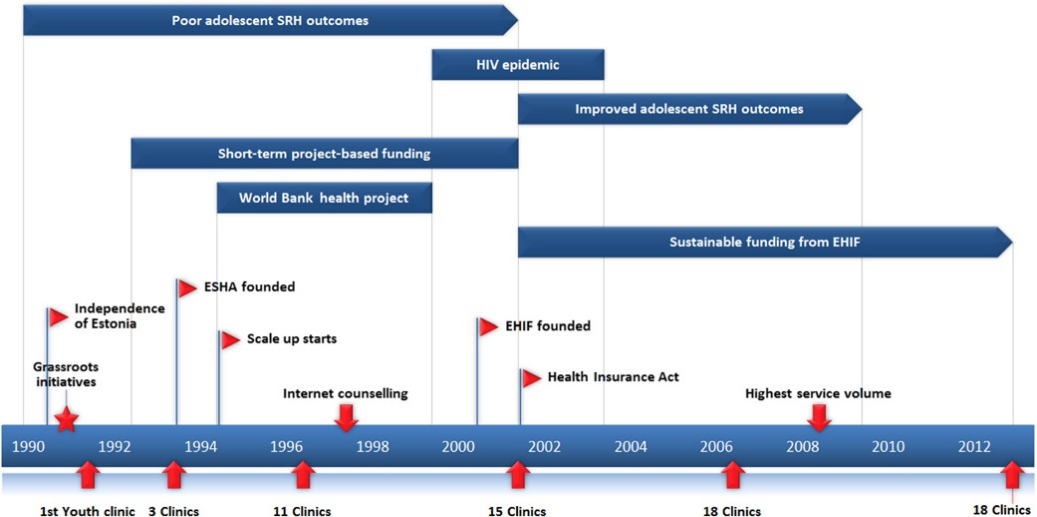
**Timeline of milestones and external factors of Estonian youth clinic network 1991–2013.**

The findings are presented in two main phases: 1) planning the scale-up strategy 1991–1995 and 2) managing the scaling-up 1996–2013. In 1996 the pace of establishing new YCs accelerated. Therefore this year was chosen as a starting point of the scale-up phase. The timeline in Figure 1 provides an overview of milestones and external factors that influenced the development and successful scale-up of the YCN.

### Planning the scale-up strategy 1991–1995

The planning strategy section covers four key elements: 1) innovation, 2) user organizations, 3) environment, and 4) resource team.

In the WHO-ExpandNet framework *innovation* refers to health service components or practices that are new or perceived as new in a particular context [1]. In this study the innovation is the *adoption of the Swedish model of delivering youth-friendly SRH services in specialized YCs*. According the framework attributes of effective innovations are; credibility, relevance, clarity, compatibility and ease of installation.

Sweden has had a national system of YCs for several decades, which added to the *credibility* of this innovation. In this model SRH services, that are confidential and free of charge, are delivered in a youth-friendly atmosphere in specialized YCs. Personnel of the YCs are trained in youth-friendly SRH services.

#### Relevance

Adoption of the innovation was a response to the lack of specialized youth-friendly SRH services in post-Soviet Estonia. The innovation targeted the high rates of unwanted pregnancy, abortion and STIs among young people in the early 1990s [1].

#### Clarity

The YCs provide SRH services to young people up to 24 years. The YCs provide also sexuality education lessons in their premises and in schools.

#### Compatibility and ease of installation

The new youth-friendly SRH services were supplementary to the existing adult focused services and provided (mainly) within the same premises.

The first YC, founded in Viljandi in 1991, was still a small local initiative, which provided only a few hours of youth-friendly SRH services per week. In the following four years four more YCs were founded in Tallinn, Tartu, Haapsalu and Paide. The SRH service package developed gradually when more YCs were established.

In the WHO-ExpandNet framework *user organizations* refer to the institutions that adopt and implement the innovation [2]. In the Estonian YCN there are three types of user organizations: 1) NGO owned free standing YCs (2 YCs), 2) hospitals (10 YCs) and 3) private practices (6 YCs) [9]. The two largest YCs in Tallinn and Tartu are owned by NGOs. In the hospital setting, YCs have separate rooms for serving adolescents, or hospitals’ out-patient clinics dedicate certain opening hours to youth. In the private sector there are gynaecological practices with specific opening hours for youth.

According to the framework the attributes of effective user organizations are; commitment, credibility and capacity.

#### Commitment

The need for the adolescent SRH services was recognized by the user organizations, because the early initiatives came from persons who worked for or with the user organizations. Moreover, new epidemiological data and comparison with neighbouring Nordic countries demonstrated the need to improve SRH of Estonian adolescents [7]. The user organizations committed to improving the situation.

#### Credibility and capacity

The new youth-friendly services were implemented mostly within existing healthcare facilities by specialized (re-)trained personnel. Moreover, in hospitals and private practices the new youth-friendly SRH services were supplementary to the existing adult focused services, which eased the cooperation. The innovation was rolled out on healthcare facility level. Therefore decision making and improving implementation capacity, i.e. personnel training and space, were done locally.

In the WHO-ExpandNet framework *environment* refers to conditions which are external to the user organization, but fundamentally affect the prospects for scaling up [2]. The main environmental factors, during the scale-up planning phase in 1991–1995, were: 1) socio-political and economic situation in post-independence Estonia, and 2) demonstrated poor SRH outcomes of youth.

There was a favourable social and political climate for implementation of the YCN in post-independence Estonia. Unlike in many other countries, there was only moderate moral, religious or political opposition to adolescent SRH interventions.

Major political and economic changes took place in Estonia in the early 1990s. The country was transformed from a centrally controlled communist system to a modern democratic and free market society. The collapse of the Soviet Union caused a deep recession in Estonia, which limited funding of the healthcare system. Consequently, the main challenges for the YC scale-up were financial.

The post-independence period was characterized by aspirations for a better future and eagerness to learn from experiences and best practices abroad and implement them locally. This was essential for initiation of new international cooperation and seizing opportunities to introduce youth-friendly SRH interventions, which had been proven successful abroad. This included adoption of the Swedish model of YCs.

In the early 1990s adolescent SRH outcomes were poor in Estonia. The main concerns at that time were: 1) low use of reliable contraceptive methods, 2) high unwanted pregnancy and abortion rates, and 3) sharply rising incidence of STIs [10, 11]. It became evident that adolescents SRH outcomes were much better in Western Europe and especially in neighbouring Nordic countries.

In the WHO-ExpandNet framework *resource team* refers to the individuals and organizations that seek to promote and facilitate wider use of the innovation [2]. In the Estonian situation the resource team refers to ESHA and international assistance.

According to the framework the attributes of effective resource organizations are; leadership, credibility and commitment.

#### Leadership

Estonian Sexual Health Association (ESHA) is a national NGO that coordinates and represents the independent YCs. Founding of ESHA in 1994 was a milestone in the expansion of the first YCs to a national YCN. ESHA has four employees and a workgroup which includes current and previous ESHA project managers, managers of the YCs and youth representatives.

#### Credibility

ESHA coordinated and monitored the YCs, developed quality standards, provided continuous training and lobbied for political commitment and funding. Moreover, *commitment* of ESHA’s and YCs’ personnel was crucial for transforming the first grassroots initiatives to a national network. Through the years, ESHA became a credible partner for the government and EHIF.

Substantial international assistance was available for improvement of adolescent SRH in Estonia. Several international organizations; the International Planned Parenthood Federation European Network (IPPF-EN), WHO Regional Office for Europe, Swedish International Development Agency (SIDA) and the World Bank (WB) and Global Fund for AIDS, Tuberculosis and Malaria (GF), supported creation of the YCN. The involvement of these international agencies increased the credibility of the innovation.

Support from IPPF-EN was particularly important. It assisted with the creation of ESHA and offered trainings, especially on management and advocacy. Moreover, it provided moral and some financial support. The memberships of IPPF-EN created an opportunity to network and cooperate with other SRH programmes in Finland, the Netherlands, Norway and Sweden.

The neighbouring countries provided models, training, material and financial assistance. Adoption of the Swedish model required training of Estonian specialists. In the beginning of the 1990s several Estonian SRH pioneers, gynaecologist, midwifes and counsellors, made study visits to YCs in Sweden and participated in youth-friendly SRH service courses in the Netherlands. Moreover, training materials were adapted and translated to Estonian. The adoption process included also other projects, for example SIDA’s "From Abortion to Contraception" in 1993–1995 [12].

### Managing the scale-up 1996–2013

This managing the scale-up section has two subsections: 1) organization and management, which covers: strategic choices for scaling up, advocacy, organizing the scaling-up process, mobilization of resources, and monitoring and evaluation, and 2) strategic planning and management of scale-up.

#### Organization and management

In the WHO-ExpandNet framework the *scaling up strategy* refers to the plans and actions necessary to fully establish the innovation in policies and programmes. The YCN was scaled up by using a combination of strategies.

**Figure 2:**
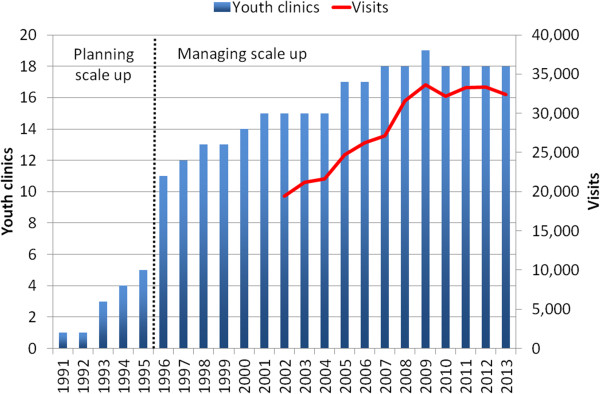
**Youth clinics in Estonia 1991–2013 and visits to the youth clinics 2002–2013.**

The primary strategy was *horizontal scaling up*, which was done by replicating YCs to new geographic areas. ESHA’s horizontal expansion approach included; 1) engagement of local decision-makers, 2) assistance with establishment and organization of new YCs, 3) training of YCs’ personnel, 4) guidelines on quality requirements and operation principles for YCs [13], and 5) acceptance of new YCs to YCN. Figure 2 illustrates the pace of horizontal scale-up YCs 1991–2013 (Kempers J: Cost Analysis of Youth Clinic Network in Estonia, forthcoming). From 1995 to 1996 the number of YCs increased from 5 to 11. In the following years the number of YCs gradually increased further to 18 in 2007, and by then the YCN was scaled up to the national level. Visit data is available since 2002, when EHIF started to centrally finance the YCs. In 2002, the YCs had 19,500 visits (excluding sexuality education lessons and internet counselling) (Kempers J: Cost Analysis of Youth Clinic Network in Estonia, forthcoming). The highest service volume was reached in 2008 (33,700 visits). Since then the number of visits stabilized at approximately 33,000 visits per year. The figure also displays the separation of: 1) planning and 2) managing scale-up phases, used in the WHO-ExpandNet framework.

*Vertical scaling up* of the YCN included institutionalization of the YCs and ESHA through: 1) ESHA and YC personnel’s’ lobby for political commitment and funding, 2) legislation change to establish EHIF [14], and 3) inclusion of the YCs in the reimbursement system of EHIF.

Also some *spontaneous scaling up* occurred, i.e. diffusion of YCs to new locations without deliberate guidance. Positive early experiences and media attention attracted enthusiasm of other SRH specialists. EHSA consolidated the spontaneous expansion by providing guidelines, training and assistance with founding and operating new YCs.

*Advocacy* was important for the successful scale-up. Personnel of ESHA and the YCs played an active role in advocating and promoting youth-friendly SRH services to policy makers and medical professionals. Repeated lobbying and fundraising efforts helped policymakers and EHIF directors to form a favourable opinion of the YCs. The need for youth SRH services was advocated by epidemiological data and many research results on adolescent SRH in Estonia. Between 1994 and 2007 there were 12 national surveys, which included adolescent sexual health items [7, 15]. At the same time national reporting systems on adolescent abortions, births and STIs were created [7]. New reliable data and results of the studies showed that Estonia was performing far worse than Western Europe and provided important advocacy materials for convincing policy makers of the need for YCs. Study results were published and presented in various meetings, which highlighted the need for special SRH services and sexuality education. In addition, the media were usually invited to opening of new YCs.

The scale-up of the YCN included a combination of *organizational approaches*. It was a multiplicative process, where ESHA built the network together with new user organizations. The "recruitment" of new user organizations for the expansion was decentralized, while the financing mechanism of the YCN became centralized. The implementation itself was a standardized expert-led process. ESHA played a key role in defining and ensuring service quality. Well-defined objectives and quality requirements were important for standardization of the scale-up. EHSA prepared a practical guideline: Quality Requirements and Operational Principles for the YCN in 2002 [13, 16, 17]. The guideline describes: 1) objectives of YCs, 2) operational principles, 3) provided SRH services, 4) target groups, 5) quality requirements, and 6) monitoring and evaluation indicators. The YCs are required to comply with the Quality Requirements and Operational Principles. The guideline has also practical materials for YCs; quality assessment checklists, job descriptions, professional recommendations, surveys and questionnaires, and a guide for founding new YCs.

*Mobilization of resources* was essential for the successful scale-up of YNC. The Estonian healthcare reform project was implemented in 1995–1999. The World Bank lent MSA 18 million USD for the project [18]. MSA itself provided an additional 14.5 million USD. Adolescent SRH and YCs are part of the wider health promotion and disease prevention policy of Estonia. The WB contract earmarked funds for prevention interventions. Women’s health and family planning were selected as one of the priority areas. Adolescent SRH services or YCs were not explicitly mentioned. Nevertheless, the loan and resulting healthcare reform enabled regional sickness funds to start financing individual YCs. Yet, the YCs still struggled with their funding during this period. The regional financing was often short-term and project-based. Moreover, the funds were limited and dependent on annual local policy priorities. Funds of the Health Project were also directly used to equip the YCs.

The deep recession in the early years of independence, was followed by 13 consecutive years of economic growth (1995–2007) [19]. Healthcare spending increased on average by 7.0% per year (2000–2009) [20]. More funds were also made available for the YCs.

In 2001 the regional sickness funds were reorganized into a single independent public agency, the *Estonian Health Insurance Fund* (EHIF) [21]. The new Health Insurance Act was passed in Parliament in 2002 [14]. This enabled EHIF to centrally finance the YCs and EHSA, as components of the national disease prevention policy. During the period 2002–2012 EHIF financed 95% of the total costs of the YCN (Kempers J: Cost Analysis of Youth Clinic Network in Estonia, forthcoming). 97% of visitors of the YCs were covered by EHIF in 2012 (Kempers J: Cost Analysis of Youth Clinic Network in Estonia, forthcoming). The centralized reimbursement system was a key factor for the sustainable scale-up of the YCN.

Some adjustments were made on EHIF’s insurance coverage during the scale-up. New laboratory tests and rapid HIV tests were added to the package. Reimbursement rates of some services were increased. However, the economic crisis in 2009 forced EHIF to lower YCs’ reimbursement rates. Since then the total budget of the 18 YCs stabilized at approximately €950,000 per year (Kempers J: Cost Analysis of Youth Clinic Network in Estonia, forthcoming).

*HIV incidence* rose sharply in Estonia in the beginning of the 2000s [22, 23]. The country had the highest rate of HIV diagnoses in Europe [24]. In response, the MSA initiated several HIV/AIDS prevention programmes, which were mainly funded by the GF. These programmes focused on high risk groups: injecting drug users and commercial sex workers. At that time the YCs did not receive significant financing from the HIV/AIDS prevention funds. However, the HIV epidemic paved the way for the sustainability of the YCN, because later the YCs were included in the Estonian National HIV and AIDS Strategy 2006–2015. This guaranteed sustainable EHIF funding for several years.

Regular reporting and *monitoring* were important for the successful scale-up of the YCN. The YCs report quarterly to ESHA. The primary use of the reporting and monitoring is management of the YCN. The information is also used for planning. The reporting includes: visitor data, medical tests and procedures, diagnosed STIs and pregnancies. Moreover, the YCs send monthly medical invoices to EHIF. The parallel reporting is used for reimbursement payments and monitoring annual YC budgets. Furthermore, ESHA reports quarterly to EHIF on activities of the YCN and services provided by the YCs.

An external *evaluation* of two YCs in 1997 encouraged further expansion of the youth-friendly SRH services [12]. In 2008 EHIF commissioned an external evaluation of the YCs [25]. The results of the evaluation were positive and the main recommendations were to: 1) continue funding of the YCN and 2) use the YCN as an example for other prevention interventions. Four surveys were carried out to assess client satisfaction of the YCs [26–29]. Moreover, young people can give feedback on YCs’ services on ESHA’s website. In addition, EHSA evaluates one to two YCs annually and gives feedback and recommendations for improvements.

The core *SRH service package* remained mainly unchanged during the scale-up process. Several additions and improvements were made: 1) HIV testing and counselling services were introduced in response to the HIV epidemic in 2000, 2) the YCN was expanded to predominantly Russian speaking areas of Estonia (Ida-Virumaa region and Narva), 3) efforts were made to increase service uptake of young men, by providing separate visiting hours for them, 4) information dissemination on availability of YCs was improved, 5) personnel shortages were addressed, 6) larger YCs started to use online appointment scheduling systems, 7) reporting of the YCs was enhanced, and 8) a website and internet counselling services were launched first in Estonian and later in Russian.

Currently the YCs provide the following SRH services [13]:

1. STI consultations; i) STI testing, treatment and follow-up consultations, ii) HIV services; voluntary confidential counselling and testing and in case of an HIV positive result referral for specialist consultation, and iii) HPV vaccination and counselling.
2. Contraceptive consultations; i) information and counselling about contraceptives, ii) prescription and renewal of prescriptions for contraceptives, and iii) insertion of contraceptive devices.
3. SRH counselling; i) psychosexual counselling , ii) pregnancy diagnostics and referral for antenatal care or safe abortion, iii) psychological counselling, and a range of iv) other SRH services, such as counselling for victims of sexual violence.
4. Sexuality education lessons at schools or YCs.
5. Website and internet counselling. On the website young people find information about puberty, intimate relationships, sexuality, pregnancy, contraceptives and STIs. The website also offers internet counselling services, where young persons’ anonymous questions about these topics are answered by personnel of the YCs.

National age group specific SRH data provided a useful means of assessing *impact* of the YCN. In the age group age 15–24 years the SRH outcomes improved remarkably during the period 2001–2009. Annual rates of abortions, STIs and diagnosed HIV infections in the age group were reduced by 37%, 55% and 89%, respectively [6, 7, 30–33]. The YCN was developed simultaneously with the Estonian SBSE programme [8]. Together the two interventions have most likely contributed to the improvement of SRH outcomes. However, the causal relationship is difficult to assess.

#### Strategic planning and management of scale-up

In the early years of the scaling up YCN *strategic planning* was hindered by a continuous struggle for financing. The gradual growth was "organic" and built on short-term funding opportunities, which exposed the YCs to shifting local priorities. After 2002, when more sustainable EHIF funding became available, EHSA’s and YCs’ *management teams* could start to focus on long-term planning. At that time the YCN was already almost scaled up to the national level (15 YCs) and there was no need for strategic scale-up planning anymore. Therefore the attention was focused on management and professionalization of the YCN. Quality of services was standardized by providing guidelines, training and technical assistance. Client centred focus was maintained with continuous trainings and repeated client satisfaction surveys. Reporting and monitoring of YCs were enhanced. Improvements and adaptations were made to the service package. Vertical scaling-up efforts were continued by lobbying for sustainable funding and political support. During the scale-up process ESHA had proven to be a strong resource team and a credible partner for the YCs, the user organizations, EHIF and policy makers.

## Discussion

What were the main factors that contributed to the successful scale-up of the YCN in Estonia? The analysis of the scale up using the WHO-ExpandNet framework highlighted the following factors:

1. Environment: Estonia had a *favourable social and political climate* for offering youth-friendly SRH services. Major political and economic changes took place in Estonia in the early 1990s, which led to the transformation of the country from a centrally controlled communist system to a modern democratic and free market society. Unlike in many other countries, there was only moderate moral, religious or political opposition to the adolescent SRH services, which was successfully overcome by EHSA and its counterparts.
2. Innovation: The *need for the innovation* was clearly *demonstrated*. In the early 1990s new epidemiological data demonstrated that adolescents SRH outcomes were much better in neighbouring Nordic countries. In response to this, the successful Swedish model of delivering youth-friendly SRH services in specialized YCs was adopted in Estonia.
3. Resource team: There is a national *professional organization that coordinates and represents the YCs*. The creation of ESHA in 1994 was a milestone in the scale-up process. ESHA also monitored the YCs, developed quality standards, provided continuous training and lobbied for political commitment and funding. Moreover, the enthusiasm and dedication of ESHA’s and YCs’ personnel were crucial for transforming the first grassroots initiatives into a national network. Through the years, ESHA became a credible partner for MSA and EHIF.

In addition, substantial *international assistance* was available for improvement of adolescent SRH in Estonia. Assistance from the neighbouring countries, i.e. providing models, training and practical and financial assistance was crucial for the development and scale-up of the YCN. This also applies to international organizations: WHO, WB, SIDA, GF and IPPF-EN. Support from IPPF-EN was particularly important. It assisted with the creation of ESHA and offered trainings, especially on management and advocacy. Further, the membership of IPPF-EN created an opportunity to network and cooperate with other SRH programmes in other countries.

1. User organizations: The *need* for the adolescent SRH services was *recognized by the user organizations* (NGO owned free standing YCs, hospitals and private practices), as the early initiatives came from persons who worked for or with the user organizations. Moreover, in hospitals and private practices the new youth-friendly SRH services were supplementary to the existing services, which made the scale-up easier.
2. Advocacy: Personnel of ESHA and the YCs played an important active role in *advocating and promoting youth-friendly SRH services* to policy makers and medical professionals. The repeated lobbying and fundraising efforts helped policymakers and EHIF directors to form a favourable opinion of the YCs. The need for youth SRH services was advocated with epidemiological data and evaluation/research results on adolescent SRH.
3. Mobilization of resources: The YCs eventually received *sustainable national funding* from EHIF. A main turning point of the YCN was the 2002 legislation change and reorganization of health insurance system, which enabled EHIF to start to centrally finance the YCs. The stable funding environment allowed ESHA and the YCs to switch their focus from short-term financial survival to long-term planning, quality improvement and sustainable scale-up of the SRH services. In addition, the economic growth in Estonia certainly influenced the sustainability of YCs’ funding.
4. Recognition of the success: Finally, *SRH outcomes among adolescents improved* remarkably during the period 2001–2009. The YCN was developed simultaneously with the Estonian school-based sexuality education programme. Together the two interventions have most likely contributed to the improvement of SRH outcomes. Measurement of the improvement would not have been possible without development of good reporting and monitoring systems. The SRH improvements have been studied and documented in many studies and international publications on adolescent SRH in Estonia. The success of the Estonian YCN is nationally and internationally recognized.

### Limitations

This framework analysis has some limitations. Firstly, this is a retrospective assessment of the scale-up process by using the WHO-ExpandNet framework. The framework was not used in planning or management of the YCN scale-up in Estonia. Secondly, in practice the planning and managing scale-up phases used in the framework analysis overlapped and were not clearly defined. Thirdly, the first developments of the YCN took place 24 years ago. At times it was challenging to find documentation or reliable recollections of the early developments.

## Conclusions

The main factors that contributed to the successful scale-up of youth-friendly sexual and reproductive health services in Estonia were: favourable social and political climate, clear demonstrated need for the adolescent services, a national professional organization that advocated, coordinated and represented the youth clinics, enthusiasm and dedication of personnel, acceptance by user organizations (YCs, hospitals and private practices) and sustainable funding through the national health insurance system. Finally, the measurement and recognition of the remarkable improvement of adolescent SRH outcomes would not have been possible without development of good reporting and monitoring systems, and many studies and international publications.

## References

[1] The World Health Organization, Department of Reproductive Health and Research (2009). Practical guidance for scaling up health service innovations.

[2] The World Health Organization, Department of Reproductive Health and Research – ExpandNet (2010). Nine steps for developing a scaling-Up strategy.

[3] The World Health Organization, Department of Reproductive Health and Research – ExpandNet (2011). Beginning with the end in mind: planning pilot projects and other programmatic research for successful scaling up.

[4] The World Health Organization (2009). Amor youth clinic network in Estonia. Analytic case studies: initiatives to increase the use of health services by adolescents.

[5] Statistics Estonia (2012). Statistical database. Population data 2012.

[6] Kivela-Kempers J, Haldre K, Part K (2013). Impact and cost-effectiveness analysis of the national school-based sexuality education programme in Estonia. Sex Education: Sexuality, Society and Learning.

[7] Haldre K, Part K, Ketting E (2012). Youth sexual health improvement in Estonia, 1990–2009: the role of sexuality education and youth-friendly services. European Journal of Contraception and Reproductive Health Care.

[8] Kivela-Kempers J, Ketting E, Baltussen R (2011). Cost and Cost-Effectiveness Analysis of School-Based Sexuality Education Programmes in Six Countries.

[9] Estonian Sexual Health Association (2013). Project plan for reproductive health counselling and STD prevention for adolescents 2013. Eesti Seksuaaltervise Liit. Projecktiplaan noorte reproduktiivtervisealane nõustamine ja seksuaalsel teel levivate infektsioonide ennetamine 2013.

[10] The World Bank (1995). Staff Appraisal Report Estonia Health Project. Report number 13297-EE.

[11] Gromyko A (1996). Sexually transmitted diseases (STDs) epidemic in eastern Europe: a call for help. Entre Nous Cph Den.

[12] Ketting E (1997). From abortion to contraception: strengthening family planning in two centres in Estonia. Final Project Evaluation.

[13] Part K, Raudsepp T, Sikk K (2010). Quality requirements and operational principles for the youth clinic network. Noorte nõustamiskeskuste tegevuse põhimõtted ja kvaliteedi juhend.

[14] Estonian Health Insurance Fund (2002). Health Insurance Act: State Gazette 2002, 62, 37.

[15] Part K, Rahu K, Rahu M, Karro H (2008). Factors associated with Estonian adolescents’ sexuality-related knowledge: Findings from the 1994 and 1999 KISS studies. Eur J Contracept Reprod Health Care.

[16] Part K, Raudsepp T, Karro H: **Quality of care in Estonian youth clinics.** *Entre Nous Cph Den* **69:**26–27.

[17] Estonian Sexual Health Association (2002). Quality requirements and operational principles for the youth clinic network. Eesti Seksuaaltervise Liit. Noorte nõustamiskeskuse tegevuse põhimõtted ja kvaliteedi juhend.

[18] The World Bank (1995). Legal ISC Files. 1995. Estonia - Health Project: Loan 3835 - Loan Agreement - Confirmed.

[19] Statistics Estonia (2013). Real GDP per capita 1995–2013.

[20] The Organisation for Economic Co-operation and Development (2013). OECD Health Data 2013. How Does Estonia Compare.

[21] The World Bank (2009). Implementation of Social Health Insurance in Estonia. A Case Study.

[22] The World Health Organization regional Office for Europe (2011). HIV Epidemic in Estonia: Analysis of Strategic Information; Case Study.

[23] Uusküla A, Kals M, Rajaleid K (2008). High-prevalence and high-estimated incidence of HIV infection among new injecting drug users in Estonia: need for large scale prevention programs. J Public Health (Oxf).

[24] European Centre for Disease Prevention and Control (2012). HIV/AIDS Surveillance in Europe 2011.

[25] Praxis Centre for Policy Studies (2008). Audit report: The Project for Reproductive Health Counselling and STI Prevention for Young People 2002–2006.

[26] Kutsar D (2000). The visitors of youth-clinics in fall 1999. Noorte nõustamiskeskuse külastaja, 1999 aasta sügistalvel.

[27] Loit M (2003). The visitors of youth clinics 2002. Report 2002. Noorte nõustamiskeskuste ja kabinettide külastajad sügisel 2002.

[28] Kutsar D (2006). The visitors of youth-clinics 2006.

[29] Lõhmus L (2007). The visitor of youth clinics 2007. Noorte nõustamiskeskuse külastaja 2007. Survey Report.

[30] National Institute for Health Development (2009). Estonian Medical Birth Registry and Estonian Abortion Registry. Live births and legally induced abortions, 1998–2009.

[31] Estonian Health Board (2009). Registered STI cases per age group, 1998–2009.

[32] Ministry of Health of Estonia (2009). HIV register of Estonia.

[33] Part K, Haldre K, Palm E (2011). The impact of sex education in the school. Haridus.

